# Sustained Release of Hydrogen Sulfide from Di(*t*-butanol)dithiophosphate Phenethylamine Salt Encapsulated into Poly(lactic acid) Microparticles to Enhance the Growth of Radish Plants

**DOI:** 10.1021/acsagscitech.2c00179

**Published:** 2022-09-01

**Authors:** Nimesh P. R. Ranasinghe Arachchige, Eric M. Brown, Ned B. Bowden

**Affiliations:** Department of Chemistry, University of Iowa, Iowa City, Iowa 52242, United States

**Keywords:** hydrogen sulfide, poly(lactic acid), slow release, dithiophosphate, radish, microparticles

## Abstract

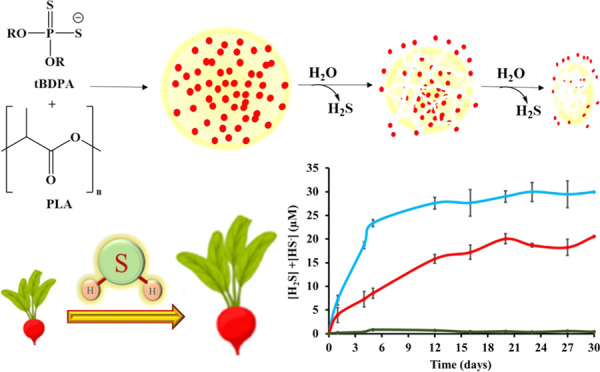

The slow release of hydrogen sulfide has been shown to be beneficial to plants by protecting them from environmental stressors, increasing germination, and extending the lifetime of harvested fruits. A major challenge in this field is controlling the amount and location of release of hydrogen sulfide so that it is available for use by plants at optimal amounts. This article reports a dual method to release hydrogen sulfide near the roots of plants by controlling its release using the hydrolysis of a dithiophosphate and the degradation of poly(lactic acid) [PLA]. Di(*t*-butanol)dithiophosphate phenylethylamine (*t*BDPA) was dissolved in a solution of PLA, and the solvent was allowed to evaporate. The resulting solid was crushed in a blender and separated into microparticles with two different size distributions of 250–500 or 500–2000 μm. The microparticles were characterized by powder X-ray diffraction to measure the presence of microcrystals of *t*BDPA within PLA, and images obtained using scanning electron microscopy with energy dispersive X-ray analysis confirmed the presence of these crystals. Microparticles of *t*BDPA loaded within PLA were characterized for their release of phosphorus and hydrogen sulfide, which both showed a burst release within 3 days, followed by a steady release. Radish plants grown with microparticles of PLA loaded with *t*BDPA had up to a 141% increase in harvest yield compared to plants grown in the presence of free *t*BDPA not loaded into PLA, PLA microparticles without *t*BDPA, and control plants grown without PLA or *t*BDPA. These experiments showed that loading hydrogen sulfide-releasing chemicals into PLA is a promising method to improve the effect of hydrogen sulfide on plants.

## Introduction

**Figure 1:**
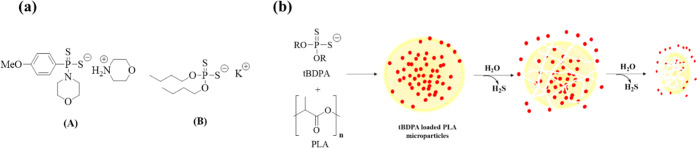
(a) Structures of GYY-4137 (A) and dibutyldithiophosphate potassium salt (B) are shown. (b) Schematic illustration to demonstrate the slow release of *t*BDPA alone with H_2_S from microparticles in the presence of water.

Hydrogen sulfide (H_2_S) is a key gasotransmitter in plants that affects numerous enzymatic pathways within, and between cells,^1−10^ and the addition of exogenous H_2_S often has a strong positive effect at surprisingly low doses on maize, soybean, wheat, cucumber, peas, tomatoes, broccoli, lettuce, sugar beets, strawberries, radishes, and kiwi plants.^6,11−24^ H_2_S is produced in cells and found at nanomolar concentrations, and recent work has shown that applying exogenous H_2_S to plants has dramatic effects, including increasing their overall size and mass, protection from high salt concentrations, protection from heat and drought stress, increased root growth, and prolonged shelf life of harvested crops.^7,21,22,25−35^ For example, the addition of exogenous H_2_S to plants such as over a dozen different plants increases their ability to withstand salt stress.^33,35^ Although the process by which exogenous H_2_S enables plants to survive salt stress is complex, it has been shown to increase the activity of antioxidant enzymes, affect the levels of Na^+^ in cells, and regulate several signaling proteins.^36−39^ The importance of exogenous H_2_S in agriculture has been established, and many current studies investigate the fundamental biochemistry of H_2_S within plants and how to apply H_2_S in a safe, dependable manner.^6^ Most of the early work in this field used micro to millimolar concentrations of aqueous H_2_S delivered once or twice daily. Although this work showed that exogenous H_2_S could improve the growth and survival of plants, most of the H_2_S evaporated due to its low boiling point of −60 °C, which led to an intense smell of H_2_S around the plants and an unknown amount of H_2_S that was adsorbed by the plants. To address these challenges, scientists used chemicals such as GYY-4137, which slowly released H_2_S by hydrolysis (Figure 1a).^40,41^ A low, one-time dose of milligrams of GYY-4137 per seed was required to have these strong, positive effects on plants.^22^ In 2019, our group demonstrated that dialkyldithiophosphates release H_2_S in water at controlled rates were stable for months in the solid state and had potential applications in agriculture. Maize grown for 4.5 weeks after exposure to 1–200 mg per seed of dibutyldithiophosphate had an increased weight of plants by up to 39% compared to control plants not exposed to dibutyldithiophosphate.^6^

Although the application of H_2_S is an exciting new frontier in agriculture, it is challenging to deliver at known doses over specified periods of time, which are two important parameters for fundamental studies of the effect of H_2_S on plants and for eventual commercial applications.^42^ Chemicals such as GYY-4137 address some of these challenges, but the rate of release of H_2_S is not easily varied from chemicals with its general structure, and it hydrolyzes to release unsafe chemicals such as morpholine that have unknown effects on plants.^43,44^ Furthermore, small chemicals such as GYY-4137 and dithiophosphates are water-soluble and can wash away from the roots of plants. What is needed in this field is a method to apply chemicals that slowly release H_2_S over well-defined time periods, release biocompatible and safe chemicals in addition to H_2_S, and will not diffuse away from the roots of plants. In this paper, we report the encapsulation of a dithiophosphate into microparticles of poly(lactic acid) [PLA] and their effect on radish plants (Figure 1b). The PLA microparticles are stationary in soil and degrade at well-understood rates.^45−49^ Dithiophosphates or other chemicals encapsulated within PLA microparticles are released at controlled rates near where the microparticles were applied within soil rather than be washed away with water. Furthermore, the degradation of PLA provides a method to provide a slow, controlled release of dithiophosphates and H_2_S near the roots of plants.

PLA was used for preparing the microparticles because it merges several interesting properties that make it an ideal candidate for our studies. PLA is a stable, odorless, and inexpensive polymer that is produced on an industrial scale.^50−52^ It naturally degrades through hydrolysis to release lactic acid and other chemicals.^46−55^ It is a key polymer in medicine to encapsulate and provide the slow release of pharmaceutical drugs. It has been approved for use in medicine, and it is used to fabricate numerous commercial products such as straws, cups, lids, landscape fabric, mulch film, and cutlery.^56−66^

In our prior work, we synthesized dithiophosphates from reactions between alcohols or thiols with P_4_S_10_ and demonstrated that these chemicals hydrolyze in water at rates that varied by a factor of over 10^4^.^44^ Dithiophosphates hydrolyzed in water to release H_2_S as well as phosphoric acid and the alcohols or thiols used in their synthesis. By selection of biocompatible alcohols or thiols, the hydrolysis products of dithiophosphates were safe for the environment and unlikely to have a strong effect on plants which allows the response of plants to H_2_S to be measured. In addition, dithiophosphate salts are solids with long shelf lives (stable over 6 months).^6^

In this paper, we describe the encapsulation of a dithiophosphate into microparticles of PLA. Encapsulation of a dithiophosphate within PLA addresses the critical problem of providing a sustained release of H_2_S near the roots of a plant because the PLA particles will remain in close proximity to where they were originally added in the soil. The microparticles have a slow, sustained release of H_2_S as the PLA degrades. In this article, we report preparation of different microparticles with different sizes and compositions, release of a dithiophosphate salt from the microparticles, and H_2_S release from microparticles in buffered water. To demonstrate the potential applications of H_2_S-releasing microparticles in agriculture, we also describe their effects on the growth of radish plants after exposure to milligram-loading of microparticles. This H_2_S delivery system provides a solution for a sustained, localized delivery of H_2_S over weeks.

## Experimental Section

### Materials and Methods

PLA [MW 150 000 g/mol] was purchased from Indeo. All other chemicals were obtained from Sigma-Aldrich at their highest purity and used as received. Nuclear magnetic resonance (NMR) spectra were obtained using a Bruker Avance-300 at 300 MHz and a Bruker DRX-400 at 400 MHz. Powder X-ray diffractometry (pXRD) was performed using a Siemens Model D5000 X-ray diffractometer (Bruker AXS Inc., WI). Scanning electron microscopy (SEM) and SEM-energy dispersive X-ray (SEM-EDX) microscopy of microparticles were performed on an S-2700 scanning electron microscopy (Hitachi, Japan). Differential scanning calorimetry (DSC) of microparticles was completed on a DSC TA Q100 instrument.

The radish seeds were purchased from Earl May Seed and Nursery. Potting mixes were obtained from Beautiful Land Products in West Branch, IA. Potting mix #4 was a peat/bark-based general-purpose growing mix and was used in these experiments. Pots were 2.5″ TEKU VCC 15 US 0600 (1.48 L) purchased from Hummert International.

### Synthesis of Di(*tert*-butanol)dithiophosphate Phenethylamine Salt (*t*BDPA)

*tert*-Butanol (0.71g, 9.54 mmol) was added slowly over 2 min to a mixture of P_2_S_5_ (0.52 g, 2.34 mmol) and THF (15 mL) under nitrogen. The contents were stirred at 45 ± 0.5 °C under a nitrogen atmosphere for 5 h using a thermostat-controlled hot plate/stirrer, and the contents were purged with pure dry nitrogen set at a pressure of 20 psi and flow rate of 10 mL/min. The excess nitrogen gas was vented via a bubbler. The contents were cooled in an ice bath, and phenethylamine (0.66 mL, 5.22 mmol) was added slowly over 2 min. The pure product was obtained after washing with hot toluene and dried under reduced pressure to give a white solid (85% yield). ^1^H NMR (400 MHz, CD_3_OD) δ 1.58 (s, 18H), δ 2.98 (t, 2H, *J* = 4.8 Hz), δ 3.19 (t, 2H, *J* = 4.8 Hz), δ 7.25–7.36 (m, 5H). ^13^C NMR (75 MHz, CD_3_OD) δ 29.40, 33.19, 40.78, 81.20, 126.76, 128.46, 128.59 and 136.80. ^31^P NMR (400 MHz, CD_3_OD) δ 93.56.

### Microparticle Fabrication Using a Modified Solvent Evaporation Technique

Microparticles with 16.7% loaded *t*BDPA in PLA (w/w) were prepared using a modified solvent evaporation technique. First, the PLA polymer (20.0 g) was dissolved in DCM (400 mL), and then methanol was added (8% v/v). *t*BDPA (4.02 g) was then added and stirred until dithiophosphate and PLA were both completely dissolved. After obtaining a clear solution, the solvent was removed under reduced pressure. The final solid was colored white. Microparticles were obtained by agitation within an Oster Classic Series blender. Next, the microparticles were separated into different sizes using sieves with different aperture sizes. Microparticles with sizes between 500–2000 and 250–500 μm were collected separately.

### Microparticle Fabrication with Phenylethylamine Hydrochloride Salt

Microparticles with 16.7% phenethylamine hydrochloride salt in PLA (w/w) were prepared using a modified solvent evaporation technique. The PLA polymer (20.01 g) was dissolved in DCM (400 mL). Phenethylamine hydrochloride (4.0 g) was added and stirred until the amine was completely dissolved. The solvent was removed under reduced pressure to yield with a white sold. Microparticles were obtained by agitation within an Oster Classic Series blender. Next, the microparticles were separated into different sizes using sieves with different aperture sizes. Microparticles with sizes between 500–2000 and 250–500 μm were collected separately.

### Scanning Electron Microscopy (SEM) and SEM-Energy Dispersive X-Ray (SEM-EDX) Microscopy of Microparticles

The shape and surface characteristics of the microparticles were investigated using SEM. The particles were placed on an aluminum specimen stub using adhesive carbon tape. The mount was then coated by ion sputtering with conductive gold set at 10 mA for 2.5 min and examined using SEM operated at a 2 kV accelerating voltage.

### Powder X-Ray Diffractometry (pXRD)

The samples were exposed to Cu Kα X-rays with a voltage of 40 kV and a current of 50 mA. The scanning angle was recorded from 10 to 80° at 25 °C at a step size of 0.020°. The scanning was obtained in a continuous scan mode.

### Differential Scanning Calorimetry (DSC) of Microparticles

The thermal behavior of microparticles was investigated by DSC. Samples were sealed in standard aluminum sample pans, and an empty sealed aluminum pan was used as a reference. Samples were purged with pure dry nitrogen set at a pressure of 20 psi and flow rate of 20 mL/min. DSC thermograms were obtained for both PLA polymer and PLA microparticles loaded with *t*BDPA by heating the samples from 30 to 180 °C with a heating rate of 10 °C/min.

### Release of *t*BDPA from Microparticles and Determination of Percentage Mass Loss from Microparticles at pH 7.2

Microparticles (0.300 g) with dimensions of 500–2000 μm and loaded with 16.7% *t*BDPA were placed in the 25 mL scintillation vials and suspended with 20 mL BIS–TRIS buffer solution (0.01 M, pH 7.2). The vials were placed on an orbital shaker at 100 rpm at room temperature and covered with an aluminum foil. Aliquots (1 mL) were taken from each vial separately at the specific time points and treated with a known concentration of methylenediphosphoric acid in D_2_O (0.016 M), which was used as an internal standard. The remainder of the vial was preserved to determine the mass loss percentage. The concentrations of *t*BDPA released into the solution were determined using ^31^P NMR spectroscopy. Each experiment was performed in triplicate.

To determine the mass loss, the remainders of the solution within the vials were used. The solution above the microparticles was pipetted out carefully, and the microparticles were dried under reduced pressure until a constant mass was obtained. This mass was compared to the initial mass of the microparticles (0.3 g). Each experiment was performed in triplicate unless stated otherwise.

### Investigation of H_2_S Release from Microparticles through a Modified Methylene Blue Reaction

To a 50 mL centrifuge tube, 0.30 g of 16.7% loaded microparticles and 10 mL of phosphate buffer solution (0.01 M, pH = 6.7) were added. A methylene blue solution (1.0 mL) was prepared in a disposable 10 mL test tube and placed inside the centrifuge tube and capped tightly. The methylene blue solution contained 0.5 mL of 0.1 M PBS (pH 6.7), 0.2 mL of 30 mM FeCl_3_ in 1.2 M HCl, 0.2 mL of 20 mM *N,N*-dimethyl-*p*-phenylene diamine in 6.7 M HCl, and 0.1 mL of 1% (w/v) Zn(OAc)_2_. The centrifuge tube was sealed and then covered with aluminum foil to avoid oxidation of H_2_S by sunlight. At selected times, the test tube was removed from the centrifuge tube, and absorbance values at 670 nm were measured. Each experiment was performed in triplicate. H_2_S release from the particles was measured by comparing to the standard curve prepared with NaHS (10, 20, 30, 40, 50, 60, and 70 μm). The same procedure was followed for 9.1% loaded *t*BDPA microparticles and 16.7% loaded phenethylamine hydrochloride microparticles.

### Growth of Radish Plants

Radish seeds were planted on October 08, 2021, in 2.5″ TEKU pots. The pots were packed finger-tight with potting mix #4 from Beautiful Land Products. The radish seeds were planted ∼1.5 in. deep. For the growth studies, 500–2000 μm-sized particles with 16.7% loaded *t*BDPA were used, and 30 seeds were planted at each loading (1, 3, 10, 30, 100, 300, and 600 mg). An additional 30 radish seeds were planted at each loading with PLA microparticles with dimensions of 500–2000 μm, and an additional 30 seeds were planted at each loading with free *t*BDPA. The amount of free *t*BDPA at each loading was calculated based on the amount of *t*BDPA within the loaded microparticles. At each loading along the *x*-axis, the amount of *t*BDPA was constant between experiments with microparticles loaded with *t*BDPA and experiments with free *t*BDPA. Radish plants were grown with 30 mg of 16.7% loaded phenethylamine hydrochloride microparticles as a control experiment. After the seeds were added to the soil, chemicals were added on the top of the seed and covered with the soil. Next, the plants were placed inside a greenhouse and watered daily. Radishes were harvested on November 12, 2021. After cutting off the shoot and brushing off the potting mix, roots were weighed on a laboratory balance.

### Statistical Analysis

Statistical analysis was performed using IBM SPSS Statistics 25. A nonparametric Kruskal–Wallis test was performed to determine the significance. Data represented are means ± standard errors, with two asterisks (**) indicating a 99% confidence interval.

## Results and Discussion

### Selection of a Dithiophosphate Salt

**Figure 2:**
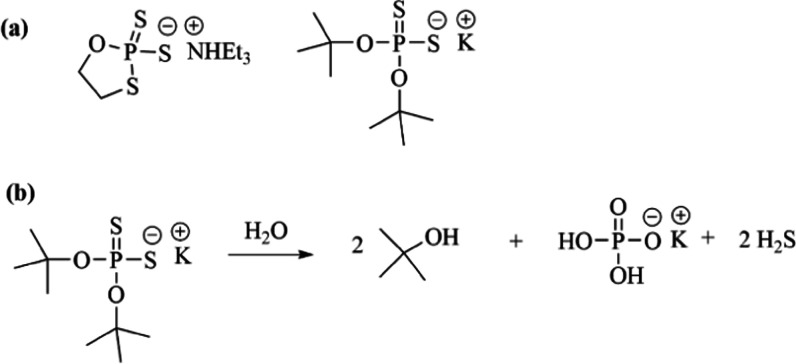
(a) Dithiophosphates synthesized from mercaptoethanol (left) and *t*-butanol (right) completely hydrolyzed in water within 30 days. (b) Complete hydrolysis of di(*t*-butanol)dithiophosphate potassium salt releases chemicals safe for the environment.

The goal of this project was to place PLA microparticles loaded with dithiophosphate in soil to slowly release hydrogen sulfide near roots to improve the growth of plants. To accomplish this goal, dithiophosphates released from the PLA particles must rapidly hydrolyze to release H_2_S before the dithiophosphates diffuse away. The first step was to select a dithiophosphate that had a rapid rate of hydrolysis, and that could be encapsulated within PLA. In a prior publication in 2021, the rates of hydrolysis of dithiophosphates synthesized from alcohols (primary, secondary, and tertiary), thiols, dithiols, and diols were investigated, and the two chemicals in Figure 2a had rates of hydrolysis that were >50× faster than the others.^44^ The dithiophosphate synthesized from mercaptoethanol was not selected for this project because mercaptoethanol is toxic, and it rapidly cleaves disulfides that are present in proteins within plant cells.

Dithiophosphates with tertiary alcohols were selected for this project due to the safety of *t*-butanol in the environment, the range of tertiary alcohols available, and the lack of odor of these alcohols. In prior work, it was shown that di(*t*-butanol)dithiophosphate potassium salt had a rapid rate of hydrolysis at room temperature in water buffered at a pH of 7.4 with a half-life of 5.7 days. Furthermore, the potassium salt was a solid and stable at room temperature for over 6 months. The complete hydrolysis of di(*t*-butanol)dithiophosphate released 2 equiv of *t*-butanol, 2 equiv of H_2_S, and phosphate salt (Figure 2b). Both phosphate (commonly used as fertilizer) and *t*-butanol are safe chemicals in the environment.^44^

**Figure 3:**
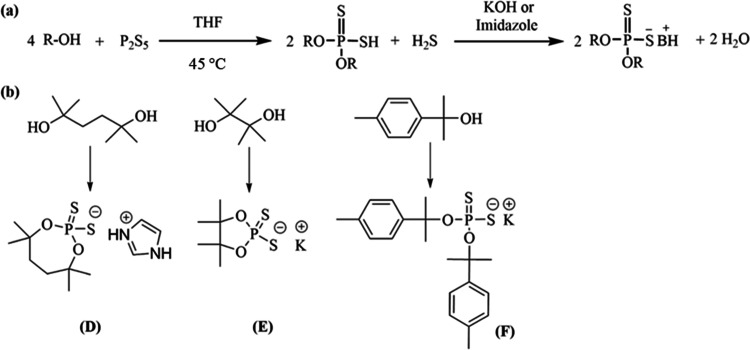
(a) General reaction scheme to synthesize dithiophosphates is shown. (b) Synthesis of **D**, **E**, and **F** are shown.

To fabricate dithiophosphates within the microparticles of PLA, it is desired to have both PLA and dithiophosphate soluble in the same solvent. The potassium salt of di(*t*-butanol)dithiophosphate was poorly soluble in methylene chloride, ethyl acetate, and acetonitrile, which are the solvents that dissolve PLA. To improve the solubility in organic solvents of dithiophosphate salts synthesized from tertiary alcohols, several dithiophosphates were synthesized, as shown in Figure 3. These chemicals were chosen because they are likely to be safe for the environment. 2,5-Dimethyl-2,5-hexanediol is a safe alcohol commonly used in perfumes, pinacol is a starting material widely used in industry that has no significant concerns outlined in its safety data sheet, and trimethylbenzyl alcohol (commonly known as cherry propanol) is found in food such as carrots, fruits, citrus, and tomatoes.^67−69^

The reactions to synthesized dithiophosphates **D**, **E**, and **F** were completed at 45 °C. The potassium and imidazolium salts were isolated to investigate their solubilities in methylene chloride, ethyl acetate, and acetonitrile. Unfortunately, both potassium and imidazolium salts of **D** and **F** were insoluble in these solvents. Moreover, hydrolysis of compound **D** in 90% H_2_O/D_2_O was investigated at 85 °C and room temperature by ^31^P NMR spectroscopy. After 30 days, only 18% hydrolyzed at 85 °C and less than 3% hydrolyzed at room temperature. The chemical **E** was soluble in dichloromethane, but its rate of hydrolysis was also slow in 90% H_2_O/D_2_O at room temperature and 85 °C, as measured by ^31^P NMR spectroscopy. Less than 3% of **E** hydrolyzed after 30 days at room temperature and only 20% hydrolyzed after 30 days at 85 °C.

**Figure 4:**
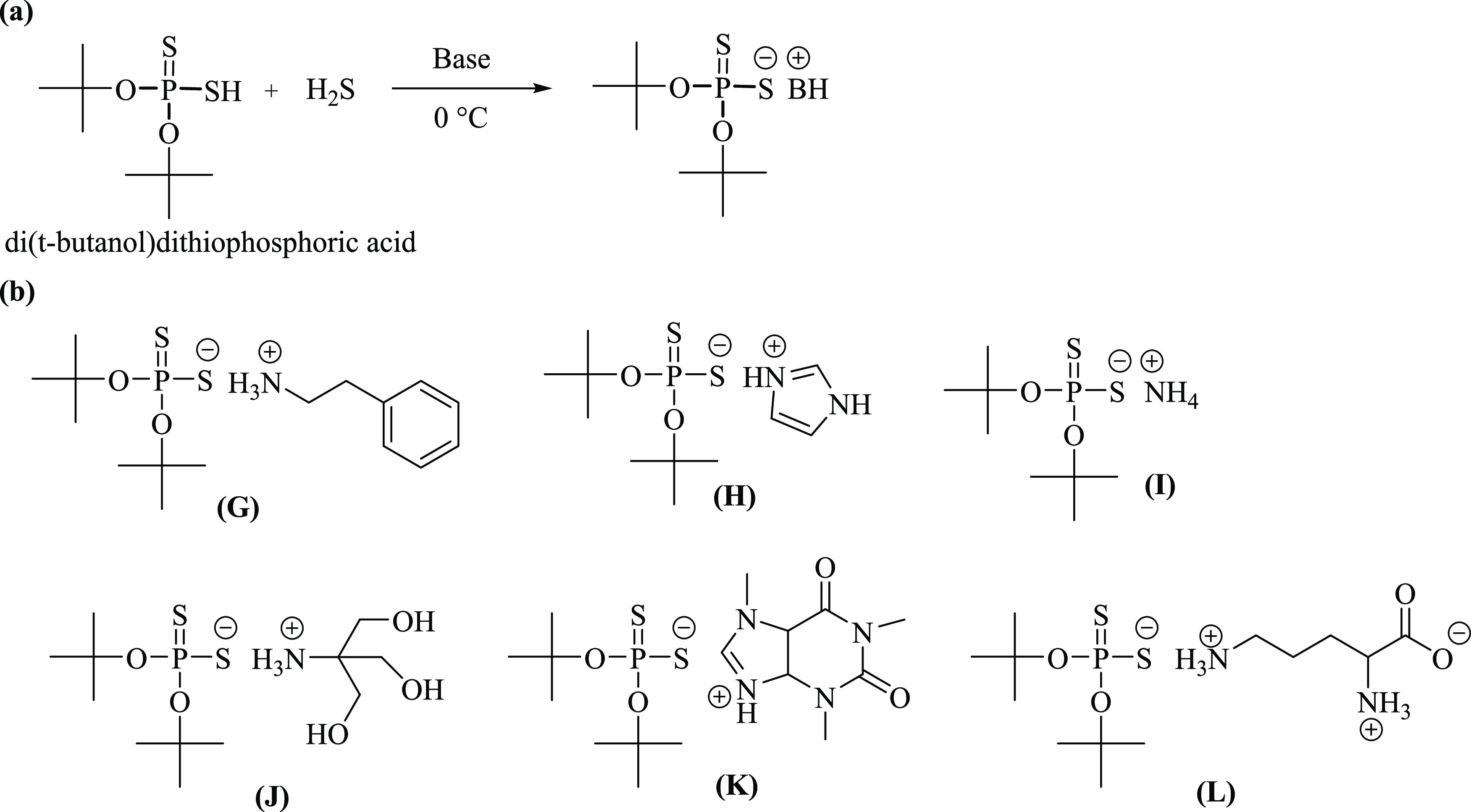
(a) General reaction scheme for the neutralization of di(*t*-butanol)dithiophosphate is shown. (b) Different salts of di(*t*-butanol)dithiophosphate are shown.

Di(*t*-butanol)dithiophosphate had the fastest rate of hydrolysis of the dithiophosphates studied,^44^ so to improve its solubility in organic solvents, different bases were used to form its salt. The choice of bases was limited to chemicals that had a history of safety in the environment and that would be expected to have little effect on plants. Of the six amines investigated, as shown in Figure 4, only the dithiophosphate **G** was soluble in methylene chloride, ethyl acetate, or acetonitrile.

The chemical **G** was synthesized by reacting di(*t*-butanol)dithiophosphoric acid with pheneythylamine. In mammals, phenethylamine is produced from the amino acid l-phenylalanine by the enzyme aromatic l-amino acid decarboxylase via enzymatic decarboxylation. In addition to its presence in mammals, phenethylamine is found in many other organisms and foods—such as chocolate—after microbial fermentation. Salts of phenethylamine are sold as dietary supplements, and it is indigested orally to improve athletic performance, depression, weight loss, mood, and attention. This data suggests that use of pheneythylamine is not hazardous and will not pollute the environment when used in low to moderate amounts.^70−73^

### Synthesis and Characterization of PLA Microparticles Containing Di(*t*-butanol)dithiophosphate Phenethylamine Salt (*t*BDPA)

PLA microparticles loaded with different amounts of *t*BDPA were prepared using a modified solvent evaporation method. The full details are described in the Experimental Section, but some of the key points are outlined here. PLA polymer (20.00 g) was dissolved in 400 mL of methylene chloride and 32 mL of methanol. The methanol was added to increase the solubility of *t*BDPA. Next, *t*BDPA (4.00 g) was added, and the solution was stirred until it was completely dissolved. The solvent was allowed to evaporate, and the PLA/dithiophosphate salt was obtained as a white solid. The solid was added to a blender and crushed to yield small particles that were separated into different-sized particles using sieves.

**Figure 5:**
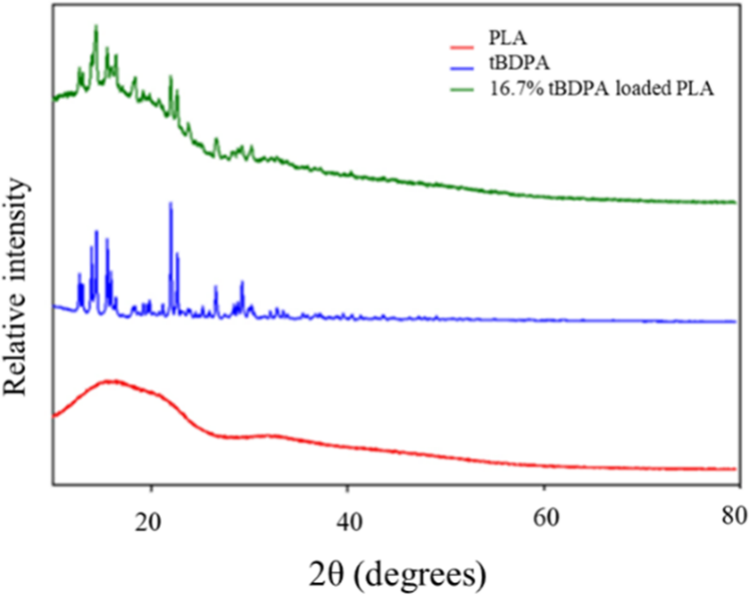
pXRD pattern of (red) PLA polymer, (blue) *t*BDPA, and (green) 16.7% *t*BDPA-loaded PLA are shown.

The composition of the loaded PLA microparticles was analyzed by powder X-ray diffraction (pXRD). The pXRD spectra of PLA, crystals of *t*BDPA, and PLA loaded with 16.7% *t*BDPA are shown in Figure 5. The pXRD spectrum of PLA was consistent with prior reports, and the pXRD spectrum of PLA loaded with 16.7% *t*BDPA showed that the PLA contained crystals of *t*BDPA.^74^ Although PLA and dithiophosphate salt were soluble in the methylene chloride/MeOH mixture, it is likely that some *t*BDPA precipitated as the solvent evaporated.

**Figure 6:**
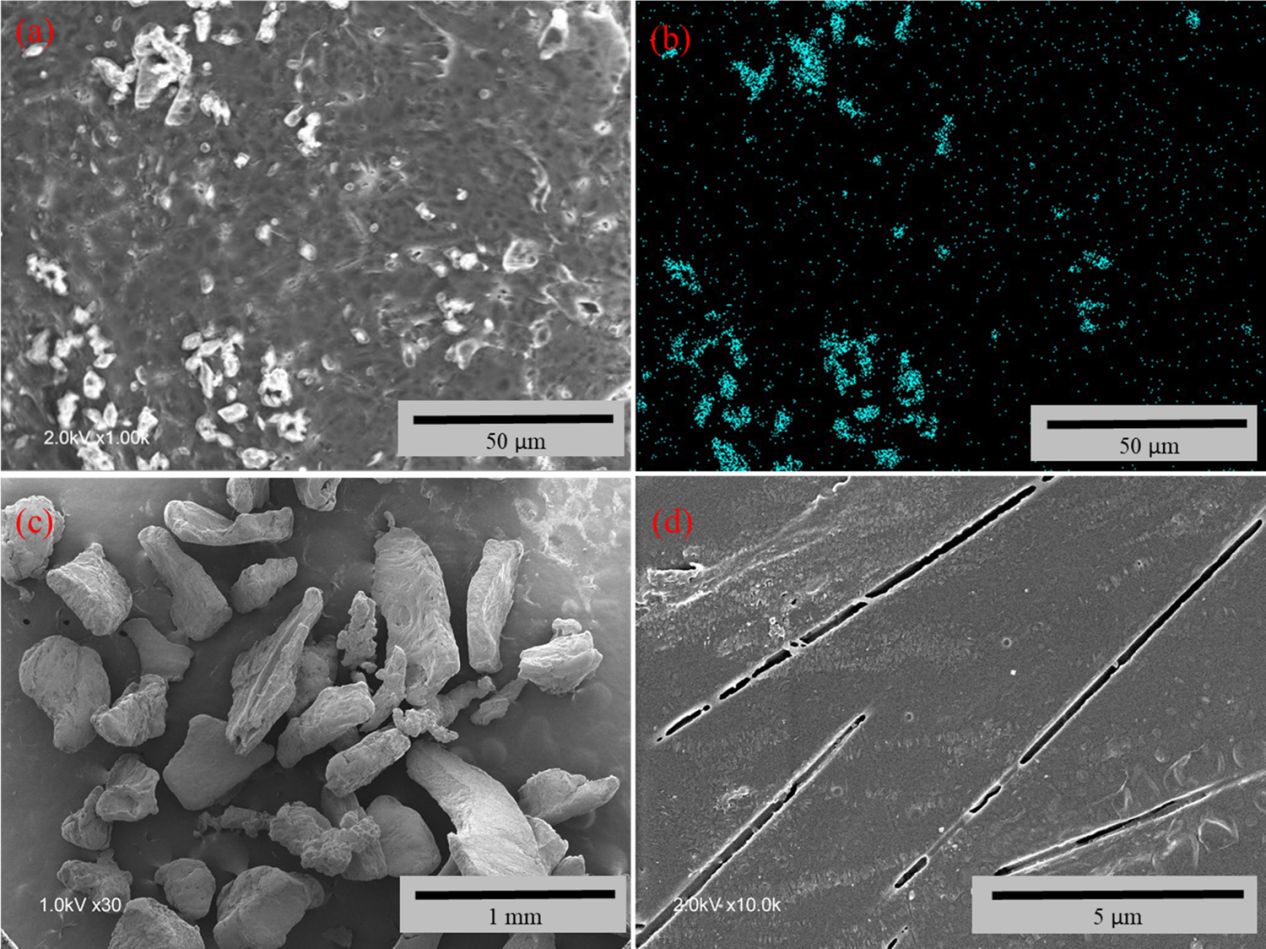
(a) Surface of PLA microparticles loaded with 16.7% *t*BDPA showed rough elements that (b) elemental mapping of sulfur demonstrated was due to microcrystals of *t*BDPA. (c) SEM micrographs of 16.7% *tert*-BDPA-loaded PLA microparticles illustrated their rough and irregular shapes. (d) PLA loaded with 16.7% *t*BDPA was immersed in water for 30 days and then removed and imaged by SEM.

The structure and composition of the microparticles were further investigated by SEM and SEM-EDX mapping (Figure 6). The PLA microparticles with sizes from 250 to 500 μm and loaded with 16.7% *t*BDPA were imaged by SEM (Figures 6c and S5). These images showed that the particles had rough edges and irregular sizes as expected based on crushing loaded PLA within a blender and separation by sieves. The loaded PLA microparticles were imaged at high magnification before exposure to water (Figure 6a,b). The surfaces appeared rough, and an SEM-EDX map showed that sulfur was concentrated in selected areas. These areas are likely due to the crystals of *t*BDPA within the PLA matrix, which was consistent with the results from the pXRD spectrum that showed crystals of *t*BDPA within PLA. Moreover, exposure of loaded PLA to water for 30 days showed fractures in the surface of the PLA (Figure 6d). These fractures may have been voids created by the release of *t*BDPA crystals from the PLA.

**Figure 7:**
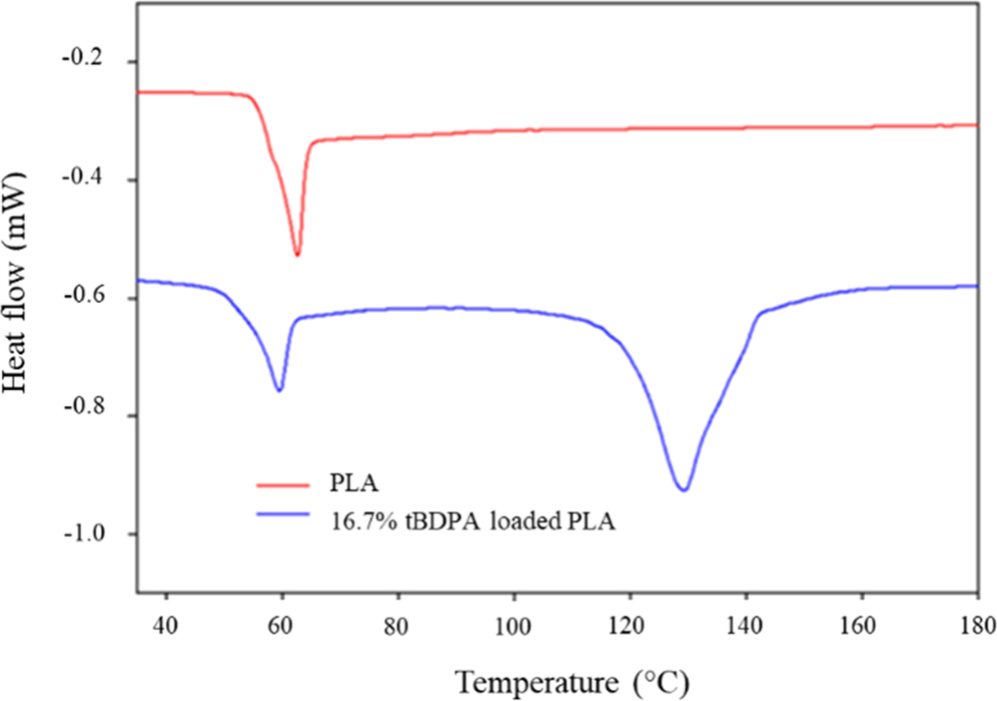
DSC thermograms of microparticles of PLA (red) and PLA loaded with 16.7% *t*BDPA (blue).

DSC thermograms of PLA microparticles and PLA microparticles loaded with 16.7% *t*BDPA were obtained (Figure 7). The DSC trace of the PLA polymer exhibited an endothermic peak, *T*_g_, at 62 °C, and this peak was observed at 56 °C for PLA loaded with the dithiophosphate salt. An additional broad peak was observed at 124 °C for PLA loaded with dithiophosphate salt that was assigned to the melting of the microcrystals of the salt. To confirm this assignment, the melting point of *t*BDPA was measured using a capillary melting point apparatus to be 130 °C.

### Release of *t*BDPA from Microparticles

**Figure 8:**
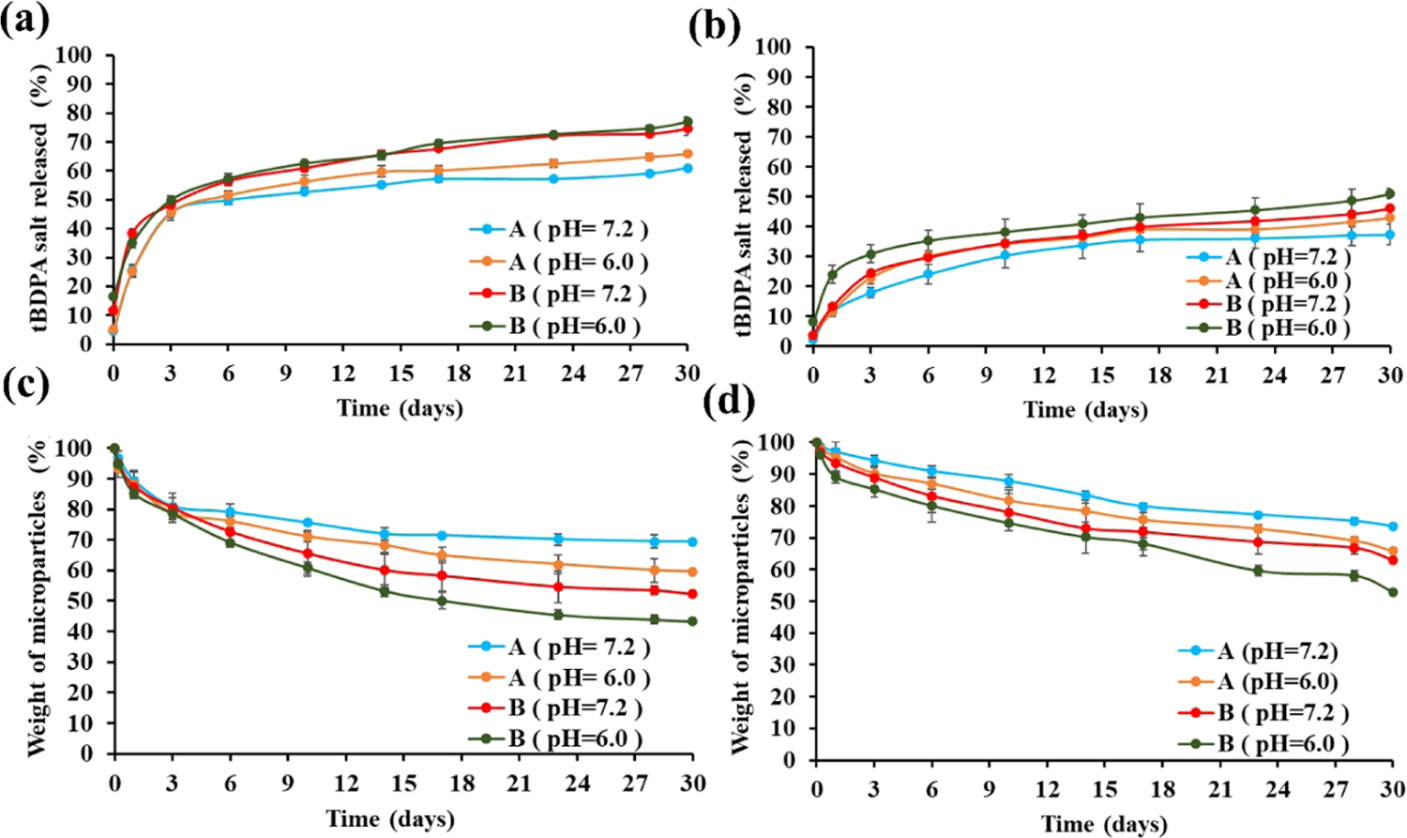
Release of phosphorous from 16.7% loaded microparticles and (b) 9.1% loaded microparticles are shown. The weight of the 16.7% loaded microparticles and (d) 9.1% loaded microparticles as a function of time are shown.

The release of *t*BDPA from microparticles was investigated in water buffered at pH values of 6.0 and 7.2. Particles with two different size distributions were selected: 500–2000 μm-sized particles and 250–500 μm-sized particles. Numerous sets of microparticles were loaded into buffered water at loadings of 15 mg/mL. The vials were constantly agitated, and at defined time points, a vial was removed, the amount of phosphorous in the buffered water was measured using ^31^P NMR spectroscopy, the microparticles were isolated and dried, and the dried microparticles were weighed. The experiments were repeated in triplicate, and the error bars for the measurements are shown in Figure 8.

The results in Figure 8 showed several important aspects of the degradation of the *t*BDPA-loaded microparticles. Microparticles loaded with 16.7% *t*BDPA had an initial rapid release of phosphate, and then a slower sustained release for both size distributions (Figure 8a). This initial rapid release was observed for other chemicals loaded into PLA in prior work.^75,76^ After 30 days, the 500–2000 μm-sized particles released 61% of the *t*BDPA at a pH of 7.2 and 74% at a pH of 6.0, and the 250–500 μm-sized particles released 66% of *t*BDPA at a pH of 7.2 and 77% at a pH of 6.0. The weight of the PLA microparticles loaded with 16.7% *t*BDPA followed a similar trend with an initial burst loss of weight, followed by a slow, steady loss of weight (Figure 8c).

PLA microparticles loaded with 9.1% *t*BDPA had a similar rapid release of phosphate within 3 days, followed by a slower release (Figure 8b). After 30 days, the 500–2000 μm-sized particles released 37 and 44% of phosphate at pH values of 7.2 and 6.0, respectively. The 250–500 μm-sized particles released 43 and 51% of phosphate at pH values of 7.2 and 6.0, respectively. In both sets of microparticles, the 250–500 μm-sized particles had a faster release of phosphate and faster loss of weight than the 500–2000 μm-sized particles at both pH values and both loadings of *t*BDPA. The more rapid release at a pH of 6.0 compared to 7.2 can be understood by the acid-catalyzed hydrolysis of PLA, which facilitated the release of *t*BDPA.^53^

### Evaluation of H_2_S Release from PLA Microparticles Loaded with *t*BDPA

**Figure 9:**
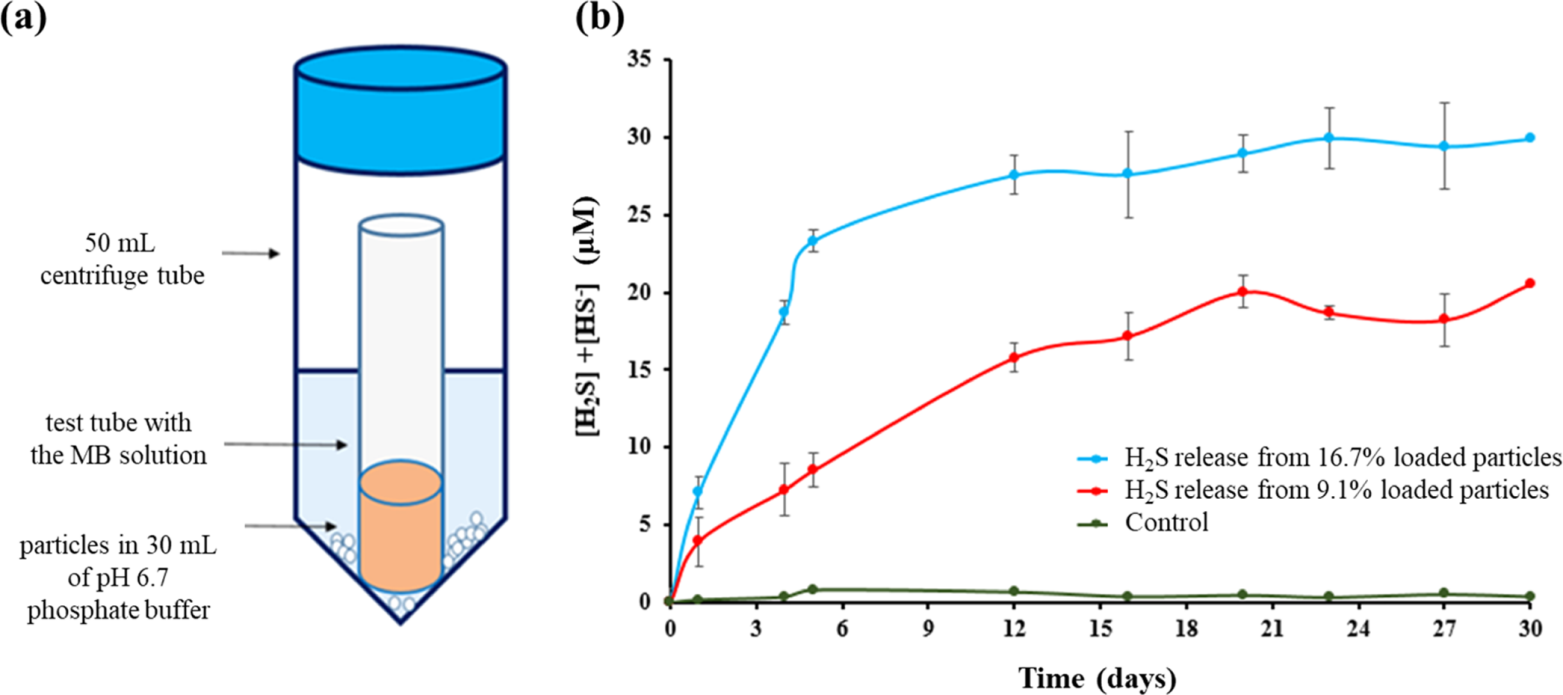
(a) Schematic of how H_2_S was trapped from the microparticles loaded with *t*BDPA is shown. (b) H_2_S release profile from 500 to 2000 μm-sized microparticles is shown.

The release of H_2_S from PLA microparticles loaded with *t*BDPA was investigated using a modified methylene blue method. The methylene blue method traps H_2_S as ZnS, which is isolated as a solid. The ZnS is then reacted with HCl, FeCl_3_, and *N*,*N*-dimethyl-*p*-phenylene diamine to yield methylene blue. The adsorption of methylene blue by UV–vis spectroscopy is then used to determine its concentration and, by extension, the amount of H_2_S that was trapped as ZnS. This method has been used for decades to report the levels of H_2_S due to its simplicity and ease of use.^77,78^ We initially investigated adding Zn(II) directly to the buffer with the loaded PLA microparticles, but a white, turbid solution was observed. To address this problem, the Zn(II) salt was added to the methylene blue solution (test tube inside the centrifuge tube), as shown in Figure 9a. When H_2_S was released from the buffer with the loaded PLA microparticles, it partitioned into the atmosphere and then into the centrifuge tube, where it was trapped by the Zn(II) to yield ZnS particles. To minimize oxidation of H_2_S promoted by light, the system was covered in Al foil.

In the experiments reported in Figure 9, 500–2000 μm-sized microparticles loaded with either 0, 9.1, or 16.7% *t*BDPA were added to water buffered at a pH of 6.7. Each experiment was repeated in triplicate, and the error bars are shown in Figure 9b. The results demonstrate that PLA without *t*BDPA did not release H_2_S as expected but that as the amount of *t*BDPA increased in the PLA, the amount of H_2_S released also increased. The microparticles loaded with 9.1% *t*BDPA had a slower, steadier increase in the amount of H_2_S released than the microparticles loaded with 16.7% *t*BDPA. The 16.7% loaded phenethylamine hydrochloride in PLA microparticles that lack *t*BDPA were used as the control. It failed to show any H_2_S releases, which demonstrated that H_2_S was released because of *t*BDPA in the microparticle.

### Growth of Radish Plants Using PLA Microparticles Loaded with 16.7% *t*BDPA

Chemicals that slowly release hydrogen sulfide have been investigated in agriculture to understand how the release of hydrogen sulfide affects their growth. Dithiophosphates are a new example of these chemicals and have been shown to increase the biomass of corn plants after 4.5 weeks of growth and to increase the harvest yield of corn by 4% after administration of a dithiophosphate once at planting of the seeds. In studies with dithiophosphates and other chemicals that slowly release H_2_S, chemicals are typically placed on seeds or in soil immediately adjacent to seeds.^44^ A challenge of chemicals used to deliver H_2_S is they are typically water-soluble and will diffuse away from plants when watered; this problem may be particularly acute after heavy rainfall. In contrast, PLA microparticles will remain stationary in soil and release dithiophosphates in the same location.

**Figure 10:**
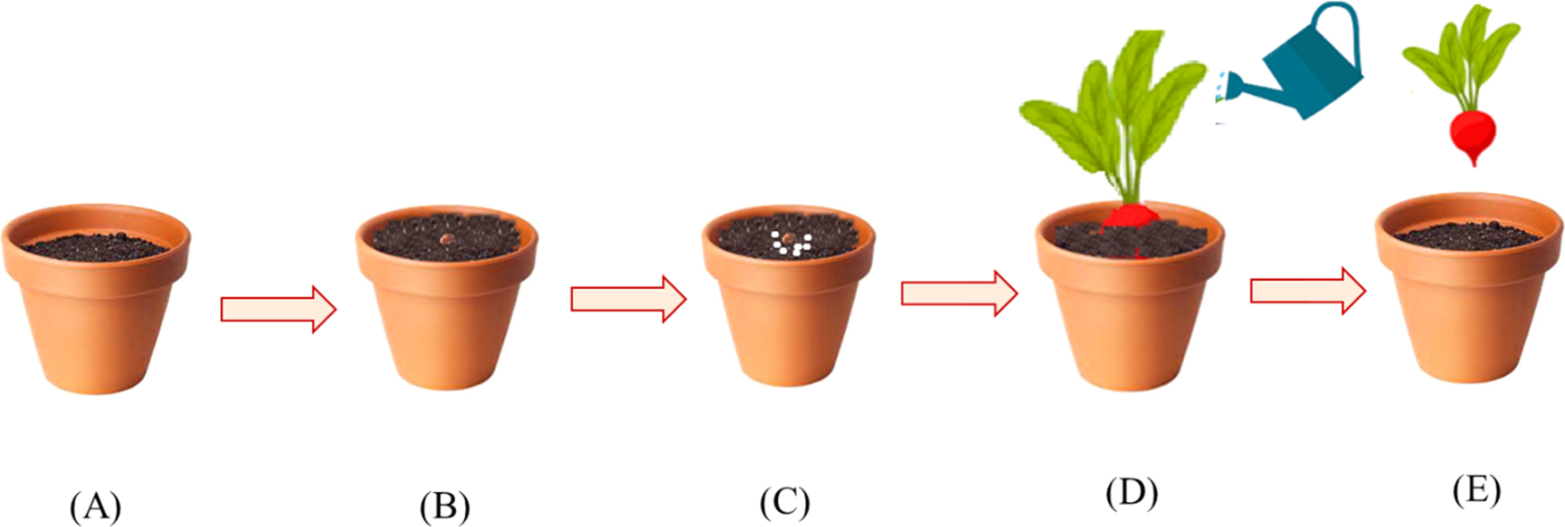
Steps for growing radish plants with different chemicals are shown. Containers were selected and filled with soil (A). Radish seeds were planted ∼1.5 in. deep (B). Different amounts of either *t*BDPA, 16.7% *t*BDPA-PLA microparticles, or PLA microparticles that did not contain *t*BDPA were added to the top of the seeds at planting and covered with the soil (C). Radish plants were watered daily (D). Radish plants were harvested after 4.5 weeks and weighed (E).

To investigate if PLA loaded with *t*BDPA could better promote the growth of plants than *t*BDPA not encapsulated within PLA, the growth of radish plants was investigated. Radish seeds were planted in individual pots and different amounts of free *t*BDPA, PLA microparticles (500–2000 μm) loaded with *t*BDPA (16.7% by weight), or PLA microparticles that did not contain *t*BDPA were added (Figure 10). The microparticles or free *t*BDPA were added to the top of the seeds at planting, and 30 seeds were planted for each loading for each additive. For instance, a total of 210 seeds were planted with PLA microparticles loaded with *t*BDPA, where the loaded PLA was planted with 1, 3, 5, 10, 30, 100, 300, and 600 mg per seed. An additional 210 seeds were planted and exposed to the same loadings of PLA not loaded with *t*BDPA.

**Figure 11:**
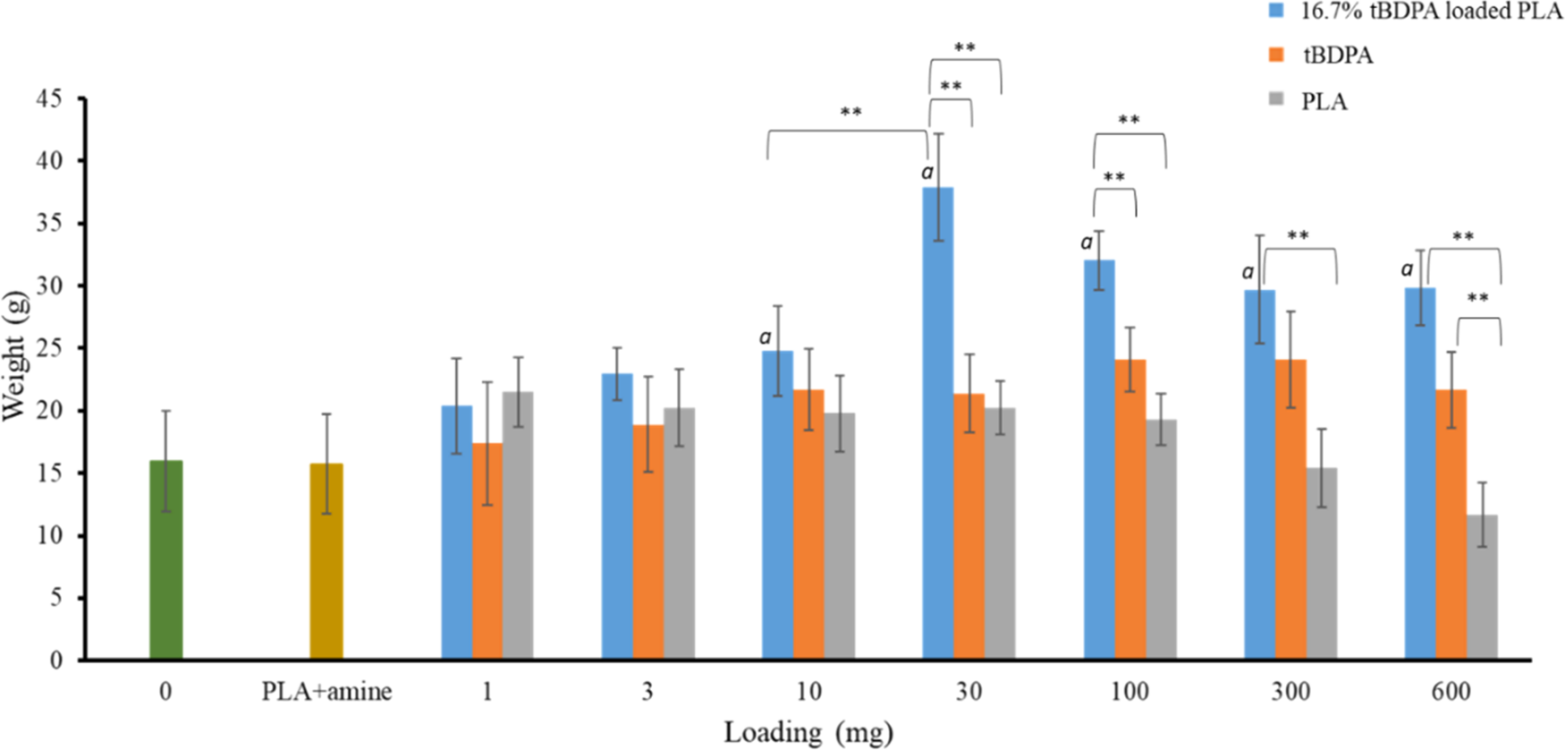
Average radish root weight of plants exposed to various doses of PLA (gray), free *t*BDPA (orange), and 16.7% *t*BDPA-loaded PLA (blue) is shown. *a* = statistical significance to zero control (radish plants without any chemicals) using the Tukey–Kramer test with a 99% confidence interval. A line connecting two groups indicates statistical significance using the Tukey–Kramer test. **99% confidence interval.

Seeds planted with PLA were investigated to determine the effect of PLA on radish plants since it was known from prior work that PLA can have a small, positive effect on plants.^79^ The loaded PLA microparticles were investigated to determine how the slow, localized release of *t*BDPA impacts the growth of radish plants. The seeds were watered daily with tap water and grown for 4.5 weeks, and then the radishes were harvested and weighed. In each of the application rates as shown in Figure 11, the weight refers to the weight of the PLA microparticles loaded with *t*BDPA. For instance, the “1 mg” loading refers to 1 mg of 16.7% *t*BDPA microparticles. The amount of free *t*BDPA added at each loading in Figure 10 was the amount of *t*BDPA within the microparticles at that loading.

The results in Figure 11 show that *t*BDPA loaded in PLA has a significant, positive effect on the growth of radish plants. The weight of the radish plants showed statistically significant improvement in growth when *t*BDPA loaded into PLA was added at loadings of 10, 30, 100, 300, and 600 mg compared to the control plants not exposed to any chemicals. The weight of the radish plants increased by 141% when only 30 mg of *t*BDPA-loaded microparticles were used per seed compared with the weight of the plants grown in the absence of these microparticles.

The radishes had the largest increase in weight when grown with PLA microparticles loaded with *t*BDPA. For all but the 1 mg application rate, radish plants grown in the presence of loaded PLA microparticles had larger weights than radishes grown in the presence of free *t*BDPA not loaded into PLA or microparticles of PLA without *t*BDPA. Application of PLA microparticles had a small, positive effect on the growth of the radishes, which is consistent with prior work that demonstrated this effect. It is likely that the degradation of PLA releases carbon into the soil that can improve the growth of radishes.^79^

In summary, we developed a method to encapsulate a dithiophosphate salt within PLA at different loadings to control the location and amount of H_2_S released near the roots of radish plants. The dithiophosphate had a rapid release of H_2_S that was complete within 30 days and hydrolyzed to release safe, natural chemicals. PLA is well known to be safe in the environment and is used in numerous agricultural products. By encapsulating a dithiophosphate within PLA, a dual method to control the release of H_2_S was developed. As the PLA hydrolyzes with water, it releases the dithiophosphate which hydrolyzes to release H_2_S. Using the degradation of PLA to slowly release dithiophosphate, the rate at which H_2_S was released to the radish plants differed from the rate of release of H_2_S from dithiophosphates not loaded into PLA. This dual method to control the release of H_2_S had a strong, positive effect on the harvest yield of radishes and points to the importance of adding control over the release of H_2_S using encapsulation. Loading dithiophosphates into PLA not only provides an extra level of control over the release of H_2_S but also allows the dithiophosphates to be released near the roots of plants. This is important because the dithiophosphates or other chemicals that are commonly used to release H_2_S are water-soluble and can diffuse away in soil particularly when the chemicals are applied early in the growth of plants. We believe that the method reported in this article will advance the investigation of the effect of H_2_S on plants.

## Supporting Information Available

Calibration curve for absorbance of methylene blue, NMR spectra of *t*BDPA, SEM micrographs of microparticles loaded with *t*BDPA (PDF)

## Notes

The authors have filed patents related to dithiophosphates, and N.B.B. has a start-up company to commercialize dithiophosphates within agriculture.

## References

[ref1] Alvarez C.; Calo L.; Romero L. C.; Garcia I.; Gotor C. An O-acetylserine (thiol) lyase homolog with L-cysteine desulfhydrase activity regulates cysteine homeostasis in Arabidopsis. Plant Physiol. 2010, 152, 656–669. 10.1104/pp.109.147975. 19955263 PMC2815857

[ref2] Riemenschneider A.; Bonacina E.; Schmidt A.; Papenbrock J. Isolation and Characterization of a Second D-Cysteine Desulfhydrase-Like Protein from Arabidopsis. In Sulfur Transport and Assimilation in Plants in the Post Genomic Era; Backhuys Publishers: Leiden, Netherlands, 2005; pp 103–106.

[ref3] Li Y.-J.; Chen J.; Xian M.; Zhou L.-G.; Han F. X.; Gan L.-J.; Shi Z.-Q. In site bioimaging of hydrogen sulfide uncovers its pivotal role in regulating nitric oxide-induced lateral root formation. PLoS One 2014, 9, e90340 10.1371/journal.pone.0090340. 24587333 PMC3937356

[ref4] Corpas F. J.; Palma J. M. H_2_S signaling in plants and applications in agriculture. J. Adv. Res. 2020, 24, 131–137. 10.1016/j.jare.2020.03.011. 32292600 PMC7150428

[ref5] Aroca A.; Gotor C.; Romero L. C. Hydrogen sulfide signaling in plants: emerging roles of protein persulfidation. Front. Plant Sci. 2018, 9, 1369 10.3389/fpls.2018.01369. 30283480 PMC6157319

[ref6] Carter J. M.; Brown E. M.; Irish E. E.; Bowden N. B. Characterization of dialkyldithiophosphates as slow hydrogen sulfide releasing chemicals and their effect on the growth of maize. J. Agric. Food Chem. 2019, 67, 11883–11892. 10.1021/acs.jafc.9b04398. 31596582

[ref7] Zhou H.; Zhou Y.; Zhang F.; Guan W.; Su Y.; Yuan X.; Xie Y. Persulfidation of nitrate reductase 2 is involved in l-cysteine desulfhydrase-regulated rice drought tolerance. Int. J. Mol. Sci. 2021, 22, 12119 10.3390/ijms222212119. 34829996 PMC8624084

[ref8] Corpas F. J.; González-Gordo S.; Palma J. M. Nitric oxide and hydrogen sulfide modulate the NADPH-generating enzymatic system in higher plants. J. Exp. Bot. 2021, 72, 830–847. 10.1093/jxb/eraa440. 32945878

[ref9] Zhang J.; Zhou M.; Zhou H.; Zhao D.; Gotor C.; Romero L. C.; Shen J.; Ge Z.; Zhang Z.; Shen W.; et al. Hydrogen sulfide, a signaling molecule in plant stress responses. J. Integr. Plant Biol. 2021, 63, 146–160. 10.1111/jipb.13022. 33058490

[ref10] Aroca A.; Gotor C. Hydrogen sulfide action in the regulation of plant autophagy. FEBS Lett. 2022, 10.1002/1873-3468.14433. 35735749

[ref11] Li T.-T.; Li Z.-R.; Hu K.-D.; Hu L.-Y.; Chen X.-Y.; Li Y.-H.; Yang Y.; Yang F.; Zhang H. Hydrogen sulfide alleviates kiwifruit ripening and senescence by antagonizing effect of ethylene. HortScience 2017, 52, 1556–1562. 10.21273/HORTSCI12261-17.

[ref12] Wang H.; Ji F.; Zhang Y.; Hou J.; Liu W.; Huang J.; Liang W. Interactions between hydrogen sulphide and nitric oxide regulate two soybean citrate transporters during the alleviation of aluminium toxicity. Plant, Cell Environ. 2019, 42, 2340–2356. 10.1111/pce.13555. 30938457

[ref13] Li Z.-G.; Xie L.-R.; Li X.-J. Hydrogen sulfide acts as a downstream signal molecule in salicylic acid-induced heat tolerance in maize (*Zea mays* L.) seedlings. J. Plant Physiol. 2015, 177, 121–127. 10.1016/j.jplph.2014.12.018. 25727780

[ref14] Zhang H.; Hu L. Y.; Hu K. D.; He Y. D.; Wang S. H.; Luo J. P. Hydrogen sulfide promotes wheat seed germination and alleviates oxidative damage against copper stress. J. Integr. Plant Biol. 2008, 50, 1518–1529. 10.1111/j.1744-7909.2008.00769.x. 19093970

[ref15] Liu Z.; Li Y.; Cao C.; Liang S.; Ma Y.; Liu X.; Pei Y. The role of H_2_S in low temperature-induced cucurbitacin C increases in cucumber. Plant Mol. Biol. 2019, 99, 535–544. 10.1007/s11103-019-00834-w. 30707394

[ref16] Singh V. P.; Singh S.; Kumar J.; Prasad S. M. Hydrogen sulfide alleviates toxic effects of arsenate in pea seedlings through up-regulation of the ascorbate–glutathione cycle: possible involvement of nitric oxide. J. Plant Physiol. 2015, 181, 20–29. 10.1016/j.jplph.2015.03.015. 25974366

[ref17] Yao G.-F.; Li C.; Sun K.-K.; Tang J.; Huang Z.-Q.; Yang F.; Huang G.-G.; Hu L.-Y.; Jin P.; Hu K.-D.; Zhang H. Hydrogen sulfide maintained the good appearance and nutrition in post-harvest tomato fruits by antagonizing the effect of ethylene. Front. Plant Sci. 2020, 11, 584 10.3389/fpls.2020.00584. 32477391 PMC7240128

[ref18] Yao G.-F.; Wei Z.-Z.; Li T.-T.; Tang J.; Huang Z.-Q.; Yang F.; Li Y.-H.; Han Z.; Hu F.; Hu L.-Y.; Hu K.-D.; Zhang H. Modulation of enhanced antioxidant activity by hydrogen sulfide antagonization of ethylene in tomato fruit ripening. J. Agric. Food Chem. 2018, 66, 10380–10387. 10.1021/acs.jafc.8b03951. 30208706

[ref19] Li S.-P.; Hu K.-D.; Hu L.-Y.; Li Y.-H.; Jiang A.-M.; Xiao F.; Han Y.; Liu Y.-S.; Zhang H. Hydrogen sulfide alleviates postharvest senescence of broccoli by modulating antioxidant defense and senescence-related gene expression. J. Agric. Food Chem. 2014, 62, 1119–1129. 10.1021/jf4047122. 24422501

[ref20] Thompson C. R.; Kats G. Effects of continuous hydrogen sulfide fumigation on crop and forest plants. Environ. Sci. Technol. 1978, 12, 550–553. 10.1021/es60141a001.

[ref21] Hu L.-Y.; Hu S.-L.; Wu J.; Li Y.-H.; Zheng J.-L.; Wei Z.-J.; Liu J.; Wang H.-L.; Liu Y.-S.; Zhang H. Hydrogen sulfide prolongs postharvest shelf life of strawberry and plays an antioxidative role in fruits. J. Agric. Food Chem. 2012, 60, 8684–8693. 10.1021/jf300728h. 22871304

[ref22] Carter J. M.; Brown E. M.; Grace J. P.; Salem A. K.; Irish E. E.; Bowden N. B. Improved growth of pea, lettuce, and radish plants using the slow release of hydrogen sulfide from GYY-4137. PLoS One 2018, 13, e0208732 10.1371/journal.pone.0208732. 30557337 PMC6296661

[ref23] Chang Y.-T.; Lin Y.-H.; Wang W.-J. Effects of the powder from hoggery desulfurization tanks on the salinity resistance of lettuce. Plants 2022, 11, 868 10.3390/plants11070868. 35406849 PMC9003075

[ref24] Lin X.; Zhang N.; Zhang Y.; Zhao Y.; Liu W.; Zhang W.; Wei G.; Zhang J.; Chen J. Interactions between hydrogen sulfide and rhizobia modulate the physiology and metabolism during water deficiency-induced oxidative defense in soybean. Authorea 2021, 10.22541/au.163308697.76950255. 36043459

[ref25] Jin Z.; Sun L.; Yang G.; Pei Y. Hydrogen sulfide regulates energy production to delay leaf senescence induced by drought stress in arabidopsis. Front. Plant Sci. 2018, 9, 1722 10.3389/fpls.2018.01722. 30532763 PMC6265512

[ref26] Ma D.; Ding H.; Wang C.; Qin H.; Han Q.; Hou J.; Lu H.; Xie Y.; Guo T. Alleviation of drought stress by hydrogen sulfide is partially related to the abscisic acid signaling pathway in wheat. PLoS One 2016, 11, e0163082 10.1371/journal.pone.0163082. 27649534 PMC5029883

[ref27] Shen J.; Xing T.; Yuan H.; Liu Z.; Jin Z.; Zhang L.; Pei Y. Hydrogen sulfide improves drought tolerance in Arabidopsis thaliana by microRNA expressions. PLoS One 2013, 8, e77047 10.1371/journal.pone.0077047. 24194857 PMC3806758

[ref28] Chen J.; Wang W.-H.; Wu F.-H.; He E.-M.; Liu X.; Shangguan Z.-P.; Zheng H.-L. Hydrogen sulfide enhances salt tolerance through nitric oxide-mediated maintenance of ion homeostasis in barley seedling roots. Sci. Rep. 2015, 5, 12516 10.1038/srep12516. 26213372 PMC4515593

[ref29] Raza A.; Tabassum J.; Mubarik M.; Anwar S.; Zahra N.; Sharif Y.; Hafeez M.; Zhang C.; Corpas F.; Chen H. Hydrogen sulfide: an emerging component against abiotic stress in plants. Plant Biol. 2022, 24, 540–558. 10.1111/plb.13368. 34870354

[ref30] Srivastava V.; Chowdhary A. A.; Verma P. K.; Mehrotra S.; Mishra S. Hydrogen sulfide-mediated mitigation and its integrated signaling crosstalk during salinity stress. Physiol. Plant. 2022, 174, e13633 10.1111/ppl.13633. 35060139

[ref31] Mishra S.; Chowdhary A.; Bhau B.; Srivastava V. Hydrogen sulphide-mediated alleviation and its interplay with other signalling molecules during temperature stress. Plant Biol. 2022, 24, 569–575. 10.1111/plb.13406. 35238126

[ref32] Mathur P.; Roy S.; Nasir Khan M.; Mukherjee S. Hydrogen sulphide (H_2_S) in the hidden half: Role in root growth, stress signalling and rhizospheric interactions. Plant Biol. 2022, 24, 559–568. 10.1111/plb.13417. 35334141

[ref33] Wang G.; Li B.; Peng D.; Zhao H.; Lu M.; Zhang L.; Li J.; Zhang S.; Guan C.; Ji J. Combined application of H_2_S and a plant growth promoting strain JIL321 regulates photosynthetic efficacy, soil enzyme activity and growth-promotion in rice under salt stress. Microbiol. Res. 2022, 256, 126943 10.1016/j.micres.2021.126943. 34953293

[ref34] Sun Y.; Song K.; Guo M.; Wu H.; Ji X.; Hou L.; Liu X.; Lu S. A NAC transcription factor from ‘sea rice 86′ enhances salt tolerance by promoting hydrogen sulfide production in rice seedlings. Int. J. Mol. Sci. 2022, 23, 6435 10.3390/ijms23126435. 35742880 PMC9223411

[ref35] Liu Y.; Wei L.; Feng L.; Zhang M.; Hu D.; Tie J.; Liao W. Hydrogen sulfide promotes adventitious root development in cucumber under salt stress by enhancing antioxidant ability. Plants 2022, 11, 935 10.3390/plants11070935. 35406914 PMC9002991

[ref36] Luo Z.; Li D.; Du R.; Mou W. Hydrogen sulfide alleviates chilling injury of banana fruit by enhanced antioxidant system and proline content. Sci. Hortic. 2015, 183, 144–151. 10.1016/j.scienta.2014.12.021.

[ref37] Christou A.; Manganaris G. A.; Papadopoulos I.; Fotopoulos V. Hydrogen sulfide induces systemic tolerance to salinity and non-ionic osmotic stress in strawberry plants through modification of reactive species biosynthesis and transcriptional regulation of multiple defence pathways. J. Exp. Bot. 2013, 64, 1953–1966. 10.1093/jxb/ert055. 23567865 PMC3638822

[ref38] Mostofa M. G.; Saegusa D.; Fujita M.; Tran L.-S. P. Hydrogen sulfide regulates salt tolerance in rice by maintaining Na+/K+ balance, mineral homeostasis and oxidative metabolism under excessive salt stress. Front. Plant Sci. 2015, 6, 1055 10.3389/fpls.2015.01055. 26734015 PMC4685665

[ref39] Deng Y.-Q.; Bao J.; Yuan F.; Liang X.; Feng Z.-T.; Wang B.-S. Exogenous hydrogen sulfide alleviates salt stress in wheat seedlings by decreasing Na+ content. Plant Growth Regul. 2016, 79, 391–399. 10.1007/s10725-015-0143-x.

[ref40] Alexander B. E.; Coles S. J.; Fox B. C.; Khan T. F.; Maliszewski J.; Perry A.; Pitak M. B.; Whiteman M.; Wood M. E. Investigating the generation of hydrogen sulfide from the phosphonamidodithioate slow-release donor GYY4137. MedChemComm 2015, 6, 1649–1655. 10.1039/C5MD00170F.

[ref41] Rose P.; Dymock B. W.; Moore P. K. GYY4137, A Novel Water-Soluble, H_2_s-Releasing Molecule. In Methods in Enzymology, Cadenas E.; Packer L., Eds.; Academic Press, 2015; Vol. 554, Chapter 9, pp 143–167. 10.1016/bs.mie.2014.11.014 25725521

[ref42] Hydrogen Sulfide. https://www.osha.gov/hydrogen-sulfide/hazards (accessed 2022-07-01).

[ref43] Feng W.; Teo X.-Y.; Novera W.; Ramanujulu P. M.; Liang D.; Huang D.; Moore P. K.; Deng L.-W.; Dymock B. W. Discovery of new H_2_S releasing phosphordithioates and 2, 3-dihydro-2-phenyl-2-sulfanylenebenzo [d][1, 3, 2] oxazaphospholes with improved antiproliferative activity. J. Med. Chem. 2015, 58, 6456–6480. 10.1021/acs.jmedchem.5b00848. 26147240

[ref44] Brown E. M.; Ranasinghe Arachchige N. P. R.; Paudel A.; Bowden N. B. Synthesis, stability, and kinetics of hydrogen sulfide release of dithiophosphates. J. Agric. Food Chem. 2021, 69, 12900–12908. 10.1021/acs.jafc.1c04655. 34694792 PMC8569798

[ref45] Vasile C.; Pamfil D.; Râpă M.; Darie-Niţă R. N.; Mitelut A. C.; Popa E. E.; Popescu P. A.; Draghici M. C.; Popa M. E. Study of the soil burial degradation of some PLA/CS biocomposites. Composites, Part B 2018, 142, 251–262. 10.1016/j.compositesb.2018.01.026.

[ref46] Weng Y.-X.; Wang L.; Zhang M.; Wang X.-L.; Wang Y.-Z. Biodegradation behavior of P (3HB, 4HB)/PLA blends in real soil environments. Polym. Test. 2013, 32, 60–70. 10.1016/j.polymertesting.2012.09.014.

[ref47] Teixeira S.; Eblagon K. M.; Miranda F.; R Pereira M. F.; Figueiredo J. L. Towards controlled degradation of poly (lactic) acid in technical applications. C 2021, 7, 42 10.3390/c7020042.

[ref48] Rosli N. A.; Karamanlioglu M.; Kargarzadeh H.; Ahmad I. Comprehensive exploration of natural degradation of poly (lactic acid) blends in various degradation media: A review. Int. J. Biol. Macromol. 2021, 187, 732–741. 10.1016/j.ijbiomac.2021.07.196. 34358596

[ref49] Li T.; Sun H.; Han H.; Zhang C.; Li B.; Huang J.; Sun D. Ultrafast bulk degradation of polylactic acid by artificially cultured diatom frustules. Compos. Sci. Technol. 2022, 223, 109410 10.1016/j.compscitech.2022.109410.

[ref50] Casalini T.; Rossi F.; Castrovinci A.; Perale G. A Perspective on polylactic acid-based polymers use for nanoparticles synthesis and applications. Front. Bioeng. Biotechnol. 2019, 7, 259 10.3389/fbioe.2019.00259. 31681741 PMC6797553

[ref51] Lunt J. Large-scale production, properties and commercial applications of polylactic acid polymers. Polym. Degrad. Stab. 1998, 59, 145–152. 10.1016/S0141-3910(97)00148-1.

[ref52] Ranakoti L.; Gangil B.; Mishra S. K.; Singh T.; Sharma S.; Ilyas R.; El-Khatib S. Critical review on polylactic acid: Properties, structure, processing, biocomposites, and nanocomposites. Materials 2022, 15, 4312 10.3390/ma15124312. 35744371 PMC9228835

[ref53] Gorrasi G.; Pantani R. Hydrolysis and Biodegradation of Poly(lactic acid). In Synthesis, Structure and Properties of Poly(lactic acid), Springer, 2018; pp 119–151.

[ref54] Elsawy M. A.; Kim K.-H.; Park J.-W.; Deep A. Hydrolytic degradation of polylactic acid (PLA) and its composites. Renewable Sustainable Energy Rev. 2017, 79, 1346–1352. 10.1016/j.rser.2017.05.143.

[ref55] Cui L.; Wang X.; Szarka G.; Hegyesi N.; Wang Y.; Sui X.; Pukánszky B. Quantitative analysis of factors determining the enzymatic degradation of poly (lactic acid). Int. J. Biol. Macromol. 2022, 209, 1703–1709. 10.1016/j.ijbiomac.2022.04.121. 35487382

[ref56] Abd Alsaheb R. A.; Aladdin A.; Othman N. Z.; Abd Malek R.; Leng O. M.; Aziz R.; El Enshasy H. A. Recent applications of polylactic acid in pharmaceutical and medical industries. J. Chem. Pharm. Res. 2015, 712, 51–63.

[ref57] Castro-Aguirre E.; Iñiguez-Franco F.; Samsudin H.; Fang X.; Auras R. Poly(lactic acid)—Mass production, processing, industrial applications, and end of life. Adv. Drug Delivery Rev. 2016, 107, 333–366. 10.1016/j.addr.2016.03.010. 27046295

[ref58] Singhvi M.; Zinjarde S.; Gokhale D. Polylactic acid: synthesis and biomedical applications. J. Appl. Microbiol. 2019, 127, 1612–1626. 10.1111/jam.14290. 31021482

[ref59] Lassalle V.; Ferreira M. L. PLA nano-and microparticles for drug delivery: an overview of the methods of preparation. Macromol. Biosci. 2007, 7, 767–783. 10.1002/mabi.200700022. 17541922

[ref60] Rancan F.; Papakostas D.; Hadam S.; Hackbarth S.; Delair T.; Primard C.; Verrier B.; Sterry W.; Blume-Peytavi U.; Vogt A. Investigation of polylactic acid (PLA) nanoparticles as drug delivery systems for local dermatotherapy. Pharm. Res. 2009, 26, 2027–2036. 10.1007/s11095-009-9919-x. 19533305

[ref61] Nagarwal R. C.; Kumar R.; Dhanawat M.; Pandit J. Modified PLA nano in situ gel: a potential ophthalmic drug delivery system. Colloids Surf., B 2011, 86, 28–34. 10.1016/j.colsurfb.2011.03.023. 21497491

[ref62] Buhecha M. D.; Lansley A. B.; Somavarapu S.; Pannala A. S. Development and characterization of PLA nanoparticles for pulmonary drug delivery: Co-encapsulation of theophylline and budesonide, a hydrophilic and lipophilic drug. J. Drug Delivery Sci. Technol. 2019, 53, 101128 10.1016/j.jddst.2019.101128.

[ref63] Destefano V.; Khan S.; Tabada A. Applications of PLA in modern medicine. Eng. Regener. 2020, 1, 76–87. 10.1016/j.engreg.2020.08.002. PMC7474829 38620328

[ref64] Chen C.; Lv G.; Pan C.; Song M.; Wu C.; Guo D.; Wang X.; Chen B.; Gu Z. Poly (lactic acid)(PLA) based nanocomposites—a novel way of drug-releasing. Biomed. Mater. 2007, 2, L1 10.1088/1748-6041/2/4/L01. 18458473

[ref65] Freeland B.; McCarthy E.; Balakrishnan R.; Fahy S.; Boland A.; Rochfort K. D.; Dabros M.; Marti R.; Kelleher S. M.; Gaughran J. A Review of polylactic acid as a replacement material for single-use laboratory components. Materials 2022, 15, 2989 10.3390/ma15092989. 35591324 PMC9100125

[ref66] Asadollahzadeh M.; Mahboubi A.; Taherzadeh M. J.; Åkesson D.; Lennartsson P. R. Application of fungal biomass for the development of new polylactic acid-based biocomposites. Polymers 2022, 14, 1738 10.3390/polym14091738. 35566907 PMC9100248

[ref67] 2,5-Dimethyl-2,5-hexanediol Market Size and Growth Research Report 2022 by Product Scope, Overview, Opportunities, Risk, Driving Force and Forecast to 2023. https://www.rfdtv.com/story/46719215/2,5-dimethyl-2,5-hexanediol-market-size-and-growth-research-report-2022-by-product-scope-overview-opportunities-risk-driving-force-and-forecast-to-2023 (accessed 2022-07-02).

[ref68] Pinacol Pinacolone Rearrangement. https://byjus.com/chemistry/pinacol-pinacolone-rearrangement/ (accessed 2022-07-02).

[ref69] Api A. M.; Belsito D.; Biserta S.; Botelho D.; Bruze M.; Burton G. A.; Buschmann J.; Cancellieri M. A.; Dagli M. L.; Date M.; Dekant W.; Deodhar C.; Fryer A. D.; Gadhia S.; Jones L.; Joshi K.; Lapczynski A.; Lavelle M.; Liebler D. C.; Na M.; O’Brien D.; Patel A.; Penning T. M.; Ritacco G.; Rodriguez-Ropero F.; Romine J.; Sadekar N.; Salvito D.; Schultz T. W.; Siddiqi F.; Sipes I. G.; Sullivan G.; Thakkar Y.; Tokura Y.; Tsang S. RIFM fragrance ingredient safety assessment, p-α,α-trimethylbenzyl alcohol, CAS Registry Number 1197-01-9. Food Chem. Toxicol. 2020, 141, 111427 10.1016/j.fct.2020.111427. 32439589

[ref70] Irsfeld M.; Spadafore M.; Pruß B. M. β-phenylethylamine, a small molecule with a large impact. WebmedCentral 2013, 4, 4409. 24482732 PMC3904499

[ref71] Lindemann L.; Hoener M. C. A renaissance in trace amines inspired by a novel GPCR family. Trends Pharmacol. Sci. 2005, 26, 274–281. 10.1016/j.tips.2005.03.007. 15860375

[ref72] Wang X.; Li J.; Dong G.; Yue J. The endogenous substrates of brain CYP2D. Eur. J. Pharmacol. 2014, 724, 211–218. 10.1016/j.ejphar.2013.12.025. 24374199

[ref73] Berry M. D. Mammalian central nervous system trace amines. Pharmacologic amphetamines, physiologic neuromodulators. J. Neurochem. 2004, 90, 257–271. 10.1111/j.1471-4159.2004.02501.x. 15228583

[ref74] Silverajah V. S. G.; Ibrahim N. A.; Yunus W. M. Z. W.; Hassan H. A.; Woei C. B. A comparative study on the mechanical, thermal and morphological characterization of poly (lactic acid)/epoxidized palm oil blend. Int. J. Mol. Sci. 2012, 13, 5878–5898. 10.3390/ijms13055878. 22754338 PMC3382784

[ref75] Stloukal P.; Kucharczyk P.; Sedlarik V.; Bazant P.; Koutny M. Low molecular weight poly(lactic acid) microparticles for controlled release of the herbicide metazachlor: preparation, morphology, and release kinetics. J. Agric. Food Chem. 2012, 60, 4111–4119. 10.1021/jf300521j. 22480233

[ref76] Juni K.; Ogata J.; Nakano M.; Ichihara T.; Mori K.; Akagi M. Preparation and evaluation in vitro and in vivo of polylactic acid microspheres containing doxorubicin. Chem. Pharm. Bull. 1985, 33, 313–318. 10.1248/cpb.33.313. 4006022

[ref77] Zheng Y.; Liao F.; Du J.-B.; Tang C.-S.; Xu G.-H.; Geng B. Modified methylene blue method for measurement of hydrogen sulfide level in plasma. Acta Physiol. Sin. 2012, 64, 681–686. 23258332

[ref78] Moest R. R. Hydrogen sulfide determination by the methylene blue method. Anal. Chem. 1975, 47, 1204–1205. 10.1021/ac60357a008.

[ref79] Chang Y.-N.; Mueller R. E.; Iannotti E. L. Use of low MW polylactic acid and lactide to stimulate growth and yield of soybeans. Plant Growth Regul. 1996, 19, 223–232. 10.1007/BF00037795.

