# Supplementary material for: Sustained Release of Hydrogen Sulfide from Di(t-butanol)dithiophosphate Phenethylamine Salt Encapsulated into Poly(lactic acid) Microparticles to Enhance the Growth of Radish Plants
Source: ACS Agric Sci Technol. 2022 Sep 1;2(5):1052–62. doi: 10.1021/acsagscitech.2c00179 (PMC10118237; doi:10.1021/acsagscitech.2c00179)
Supplement: Supplementary file 1 — as2c00179_si_001.pdf [file as2c00179_si_001.pdf]

Supporting Information

Sustained Release of Hydrogen Sulfide from Di(*t*-butanol)dithiophosphate Phenethylamine Salt Encapsulated into Poly(lactic acid) Microparticles to Enhance Growth of Radish Plants

Nimesh P.R. Ranasinghe Arachchige, Eric M. Brown, Ned B. Bowden*

Department of Chemistry, University of Iowa, Iowa City, Iowa, 52242, United States of America

ned-bowden@uiowa.edu

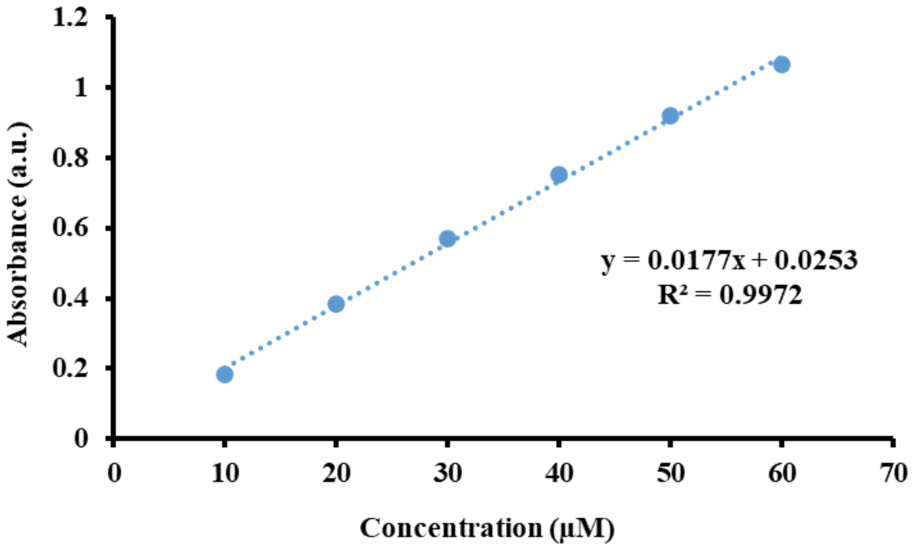

Figure S1. The calibration curve for absorbance of methylene blue by using known concentrations of H_2S.

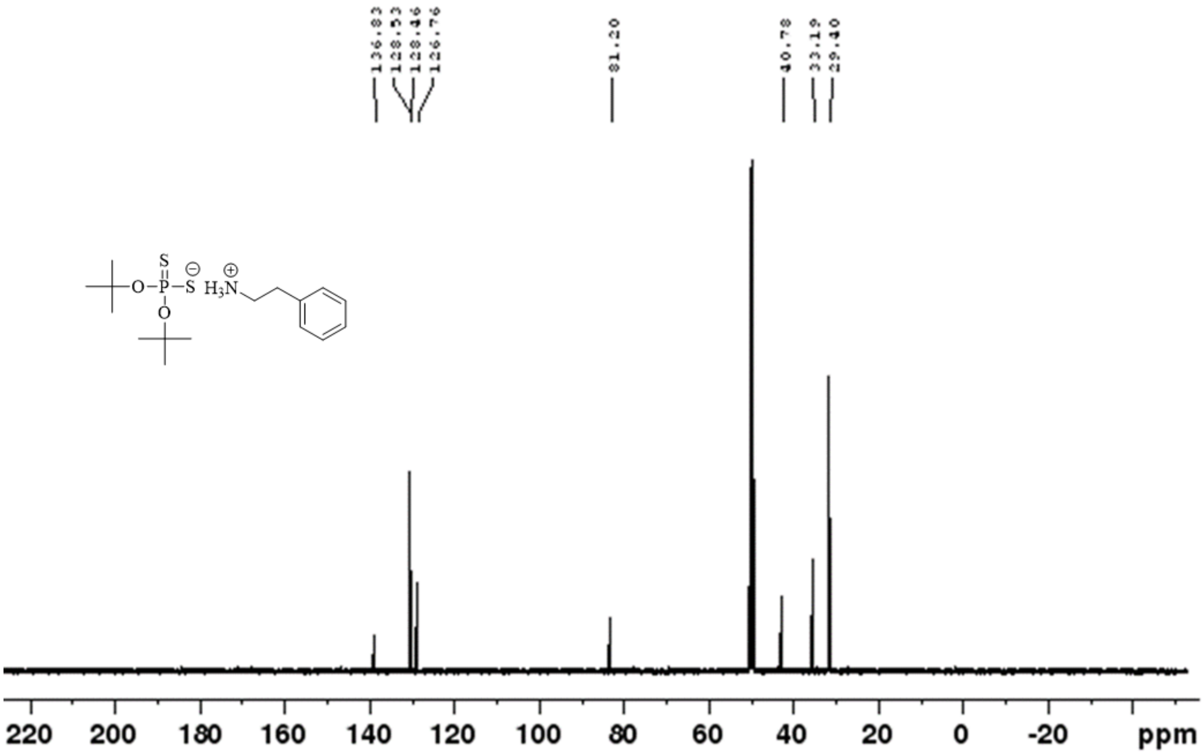

Figure S2. ^{13}C NMR spectrum of the tBDPA is shown.

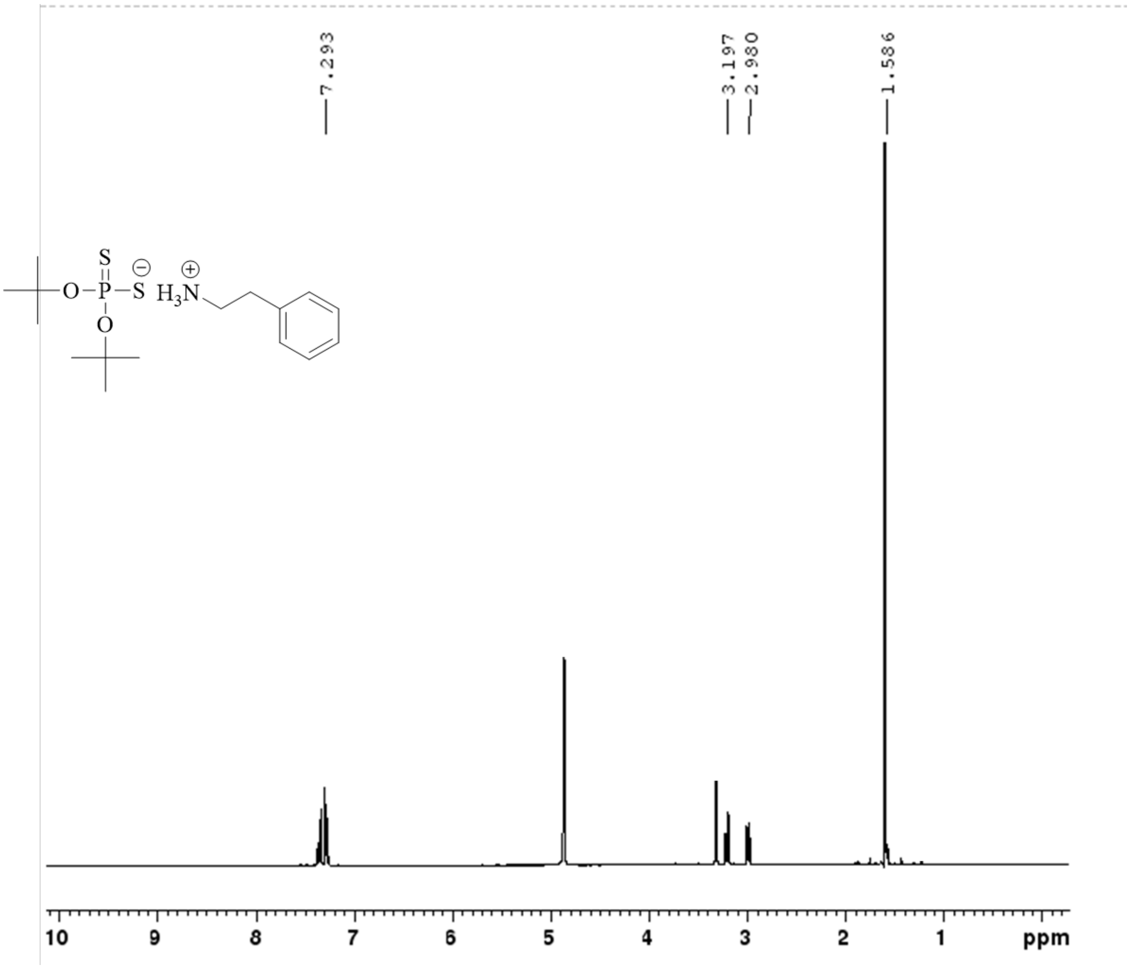

Figure S3. ^{1}H NMR spectrum of the tBDPA is shown.

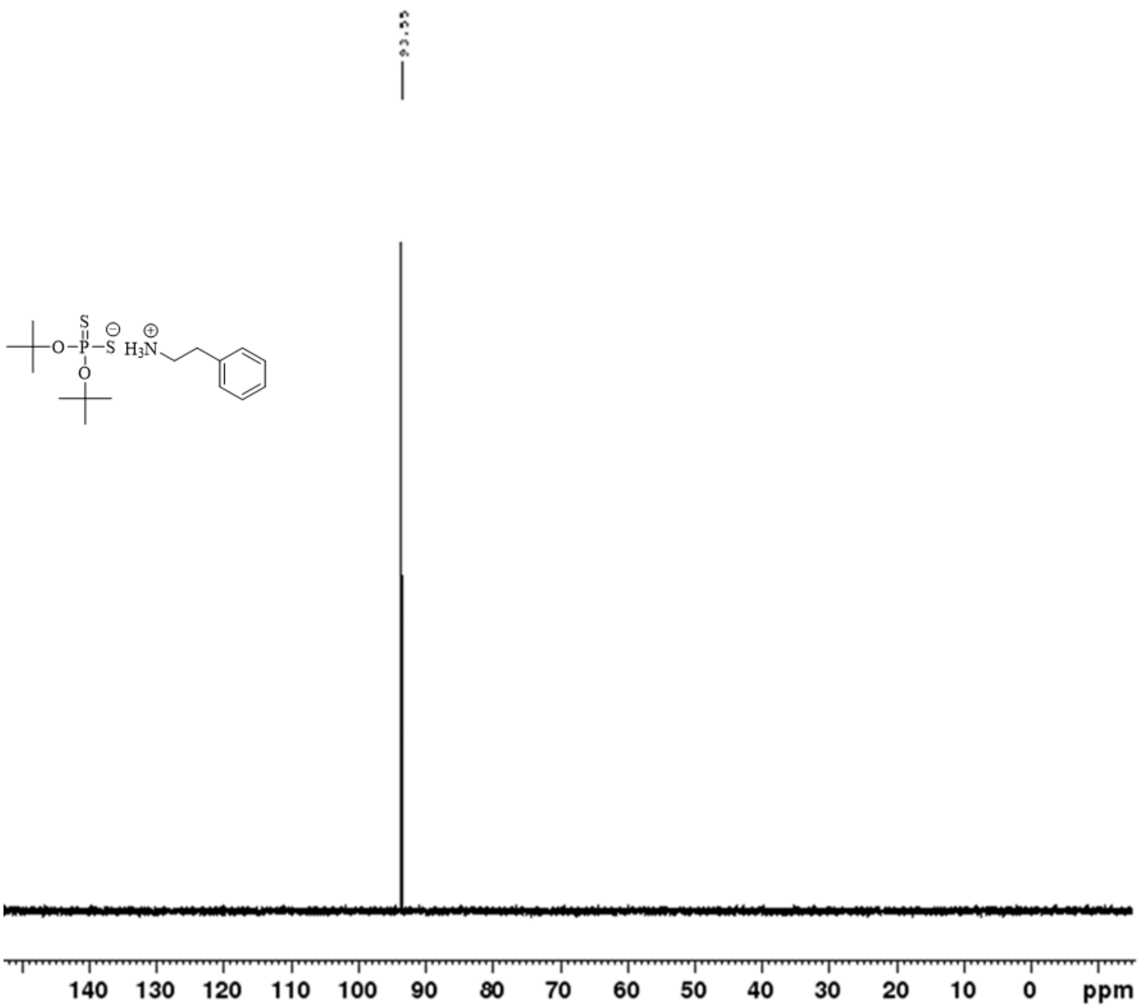

Figure S4. ^{31}P NMR spectrum of the tBDPA is shown.

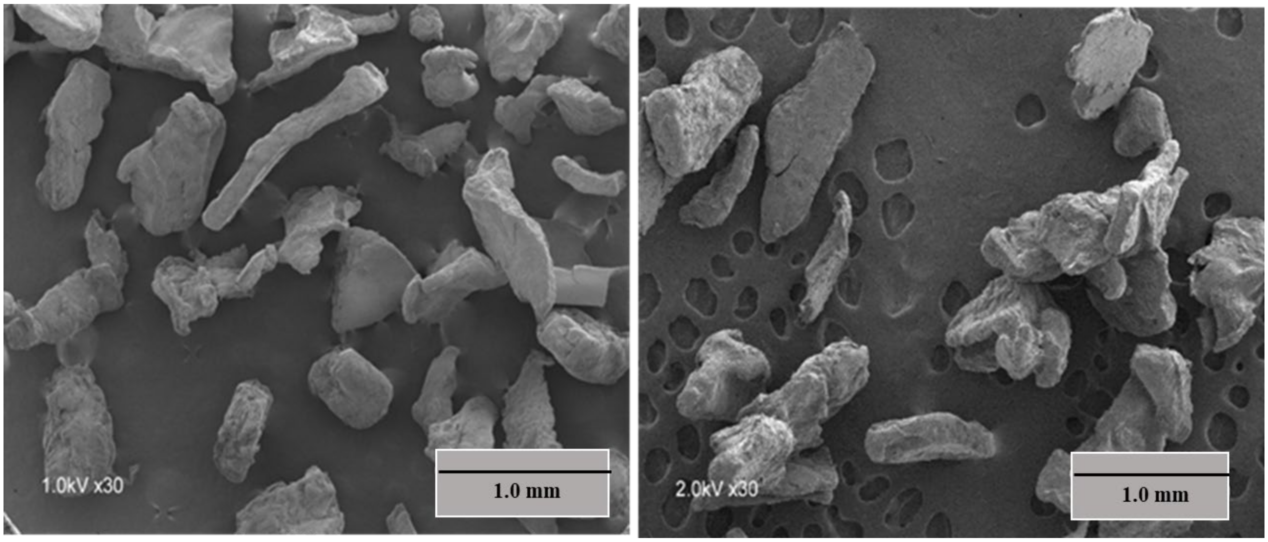

Figure S5. SEM micrographs of 500-2000 micron sized, 16.7% tBDPA loaded PLA illustrates its rough and irregular shape.

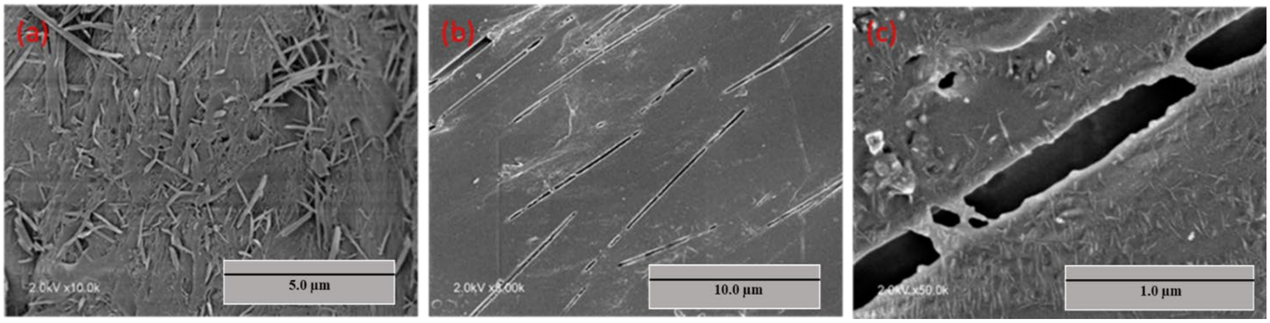

Figure S6. (a) The surface of PLA microparticles loaded with 16.7% tBDPA showed rough areas which were attributed to tBDPA crystals. (b) SEM micrograph to illustrate the fractures in the surface of the PLA, after expose to water for 30 days and (c) these fractures were imaged under higher magnification.
